# Hepatitis C Virus as a Unique Human Model Disease to Define Differences in the Transcriptional Landscape of T Cells in Acute versus Chronic Infection

**DOI:** 10.3390/v11080683

**Published:** 2019-07-26

**Authors:** David Wolski, Georg M. Lauer

**Affiliations:** Liver Center at the Gastrointestinal Unit, Department of Medicine, Massachusetts General Hospital and Harvard Medical School, Boston, MA 02114, USA

**Keywords:** viral hepatitis, hepatitis C virus, T cells, transcriptional regulation, transcription factors, metabolism, nucleosome

## Abstract

The hepatitis C virus is unique among chronic viral infections in that an acute outcome with complete viral elimination is observed in a minority of infected patients. This unique feature allows direct comparison of successful immune responses with those that fail in the setting of the same human infection. Here we review how this scenario can be used to achieve better understanding of transcriptional regulation of T-cell differentiation. Specifically, we discuss results from a study comparing transcriptional profiles of hepatitis C virus (HCV)-specific CD8 T-cells during early HCV infection between patients that do and do not control and eliminate HCV. Identification of early gene expression differences in key T-cell differentiation molecules as well as clearly distinct transcriptional networks related to cell metabolism and nucleosomal regulation reveal novel insights into the development of exhausted and memory T-cells. With additional transcriptional studies of HCV-specific CD4 and CD8 T-cells in different stages of infection currently underway, we expect HCV infection to become a valuable model disease to study human immunity to viruses.

## 1. Introduction

Infection with hepatitis C virus (HCV) is unique among chronic viral infections in humans because a significant proportion of newly infected persons (~20%–30%) can completely eliminate the virus, typically within six months of exposure [1]. This observation supports the prospect of an HCV vaccine that can at least prevent chronicity and its harmful sequelae, such as end-stage liver disease and cancer, if sterilizing immunity is not feasible. It also makes the first six months of HCV infection a powerful and one-of-a-kind human model system to define and directly compare immune responses that do and do not control infection within the same basic parameters.

In addition to the dichotomous outcome of HCV infection, recent introduction of direct-acting antiviral drugs (DAA) has created another unique feature to utilize HCV infection as a human paradigm [2]. As the only chronic viral infection in both humans and animals that can be cured through a highly specific targeted small molecule intervention, we can now study the effects of removing the root cause of immune exhaustion (i.e., chronic antigenic stimulation) from the host-pathogen interaction.

Despite these unique opportunities for basic investigations into human immunology, our knowledge base regarding molecular regulation of successful and failing immunity against HCV remains limited. This is largely due to technical challenges, such as extremely small populations of HCV-specific T-cells in the blood [3] due to compartmentalization of activated immune cells to the site of infection, the liver [4]. However, several recent technological breakthroughs now enable analysis of HCV-specific immunity with unprecedented depth and comprehensiveness. Therefore, HCV infection could be a model human disease to understand key differences between protective and failing immunity.

## 2. T Cells and the Outcome of HCV Infection

Both HCV-specific CD4 and CD8 T-cells are indispensable to control HCV infection. In chimpanzees, depletion of CD4 T-cells before re-infection after previous spontaneous clearance of HCV results in low-level persistence of viremia and eventual selection of viral mutations, leading to escape from the CD8 response [5]. When CD8 T-cells are deleted, HCV replication is not even partially controlled until the depletion wanes off and HCV-specific CD8 T-cells re-emerge [6]. This strong experimental evidence from an animal model of HCV infection, which has since been abandoned for ethical reasons [7], is further corroborated by studies in humans early after HCV exposure [8,9].

Such studies widely agree that a chronic outcome of infection is directly linked to an early defect in the HCV-specific CD4 response, as proliferative responses to HCV proteins are almost universally absent even during the earliest phase of the CD4 response in patients with persisting viremia [9,10]. However, this is not based on a lack of HCV-specific CD4 T-cells, which can typically be detected at least temporarily in all subjects using function-independent assays, such as HLA class II tetramers [10]. Once chronic viremia is established, HCV-specific CD4-T cells are often completely absent or reduced to extremely small populations that are barely detectable even with the most sensitive approaches [11].

In the CD8 response, HCV-specific CD8 T-cells are also detectable in most subjects early on [12], typically at higher frequency than their CD4 counterparts [13], and their appearance in early infection often coincides with some level of control of HCV viremia [8]. After a phase that can last several months—in which virus and CD8 T-cells seem to be engaged in a continuous tug of war—there is either full and sustained control of viremia or persistent viremia with high levels of viral replication. In the latter scenario, there are signs of progressive T-cell exhaustion on HCV-specific T-cells [14] as well as the appearance of viral escape mutations in CD8 epitope regions that lead to diminished recognition of the new circulating quasispecies [15,16]. Which of these two viral escape mechanisms initiates earlier or is more important for viral persistence and whether they are a cause or consequence of viral persistence are currently unknown.

## 3. Hurdles to Deeper Analysis of HCV-Specific T-Cells

While HCV infection presents unique opportunities to study T cell responses in different disease outcomes in humans, there are also significant hurdles to analyze HCV-specific CD4 and CD8 T-cells—the most important being low frequency of these populations in the blood. Especially in chronic infection, very few specificities can usually be detected directly ex vivo, and even the most dominant HCV-specific CD8 response rarely exceeds 0.1% of peripheral CD8 T-cell populations, with most populations much lower in frequency [12,17]. This is in stark contrast to other chronic viral infections, such as human immunodeficiency virus (HIV), Epstein-Barr virus (EBV), and cytomegalo virus (CMV), in which typically many robust responses can be identified that together represent >10% of the total CD8 T-cell pool [18].

Given that until recently even the most advanced technologies to study cellular regulation on the molecular level (e.g., microarrays, assays for transposase-accessible chromatin using sequencing ATAC-seq) required thousands or more cells to deliver robust high-quality results, there was limited opportunity to take advantage of the explanatory potential of the HCV infection model. However, more powerful next-generation sequencing technologies now allow studies on the single cell level, and HCV investigators have learned to use magnetic bead enrichment of tetramer-binding HCV-specific T-cells for isolation of even the smallest T-cell populations [19]. This now allows analysis of HCV-specific CD8 and, in a subgroup of patients, CD4 T-cells during most stages of infection, including chronicity.

## 4. Transcriptional Analysis of HCV-Specific CD8 T-Cells During Early HCV Infection

**Figure 1:**
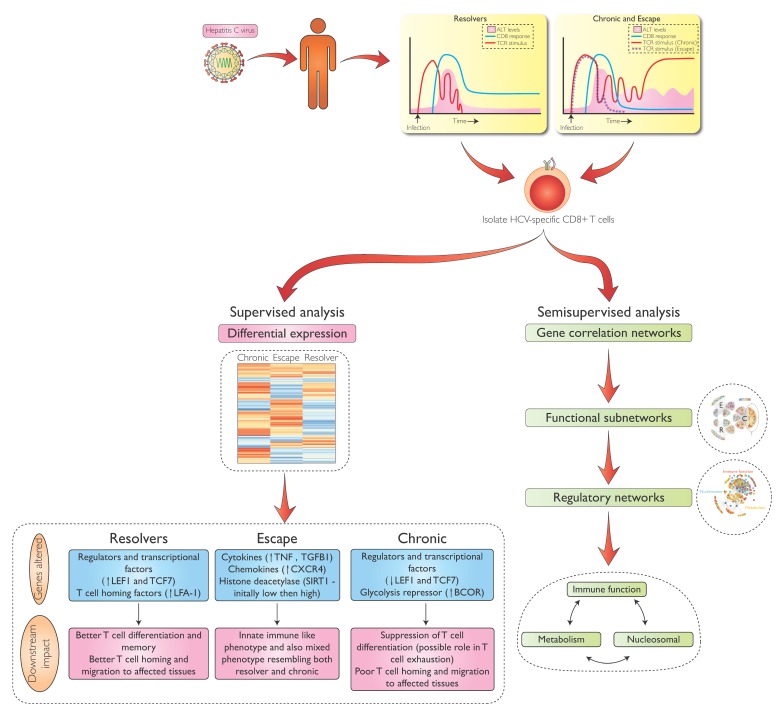
Flow diagram for the generation of transcriptional data from HCV-specific CD8 T cells in acute HCV infection followed by the different approaches to analysis of the data (as in Wolski et al., Immunity 2017).

Based on the interest in determining correlates of both protective and failing immunity and the observation that HCV-specific CD8 T-cells are detectable in most patients during the initial 6 months of infection at relatively high frequencies, we focused our first direct ex-vivo transcriptional analysis on CD8 T-cells during early HCV infection. In a recently published study [20], we analyzed HCV-specific CD8 T-cells for differential expression between groups of patients with resolving infection (Resolver) or chronic infection (see Figure 1). In chronic infection, we further distinguished between T cell populations that target the currently circulating HCV strain (Chronic) versus those in which circulating virus is dominated by viral variants not recognized by isolated HCV-specific CD8 T-cell populations (Escape). This allowed us to analyze HCV-specific CD8 T-cells from the same host that do or do not receive T cell receptor (TCR) signal and thus potentially discriminate between the impact of very specific and prolonged antigenic stimulation and non-specific consequences of a general chronic inflammatory environment in chronic infection.

## 5. Differential Gene Expression in HCV-Specific CD8 T-cells During Acute and Chronic Infection

During the initial 36 weeks of HCV infection, we identified sets of genes with both similar and differential gene expression between different outcome groups (Resolver, Chronic, Escape), using supervised differential gene expression analysis (Figure 1). Composition of these gene sets changed from the early phase (0–18 weeks) to the subsequent phase (19–36 weeks) of acute infection. For example, cells from all three groups expressed classic markers of T cell activation, such as *CD74* and *HLA-DRB1*, at high levels during the initial phase of infection, followed by later downregulation. This reduced activation of the T cell response, irrespective of outcome, is consistent with the clinical course of persistent HCV infection—inflammation is much less pronounced in the later phase of infection compared to the early acute phase, even in chronic outcomes with continued high viral replication.

In contrast to consistent transcriptional patterns across different outcomes, we identified sets of genes that were predominantly up- or downregulated in one group compared to the others. For example, Escape samples had much higher expression of the cytokines tumor necrosis factor α (*TNF)* and transforming growth factor β1 (*TGFB1)*, their respective downstream signaling components nuclear factor NF-kappa-B p105 subunit (*NFKB1)*, jun-B (*JUNB)*, krueppel-like factor 10 (*KLF10)*, and mothers against decapentaplegic homolog 7 (*SMAD7)*, as well as the chemokine receptors C-X-C chemokine receptor type 4 (*CXCR4)* and C-C chemokine receptor type 7 (*CCR7)*, compared to lower expression levels in both Chronic and Resolver groups. All of these functionally related genes continued to be expressed in Escape samples at later stages of infection. Because *TNF* and *TGFB1* signaling pathways are not normally associated with classical CD8 T-cells, our interpretation of this data is that T cells exposed to a chronic inflammatory environment without concomitant TCR stimulation adopt a more innate-like immune phenotype, a phenomenon previously described in the context of tumor immunology and hepatitis A [21,22,23]. Inclusion of Escape samples therefore adds an additional dimension to the complexity of T cell differentiation, especially given the observation of similar phenotypes in cancer, another disease in which T-cell exhaustion and dysregulation play a major role in disease pathogenesis. Differences in directly antigen-mediated and antigen-independent effects on CD8 T-cell regulation and differentiation warrant more detailed investigation.

The most important differences between outcome groups were for key genes linked to cellular differentiation programs related to T cell memory. In patients with acute resolving HCV infection, CD8 T-cells had early high expression of regulators of T cell differentiation and memory, *TCF7* and its transcriptional target *LEF1*. Both *TCF7* and *LEF1* also possess intrinsic histone deacetylase (HDAC) activity, which is thought to be functionally crucial to their control of T cell lineage commitment [24]. Both *TCF7* and *LEF1* are expressed at low levels in Chronic samples throughout early and late acute phases of infection, suggesting that T cell differentiation might be negatively impacted very early during chronic infection. Interestingly, Escape samples exhibit a mixed expression profile for these genes, sharing higher levels of *TCF7* expression similar to Resolver samples and lower levels of *LEF1* expression similar to Chronic samples, suggesting that they develop a semi-differentiated phenotype that could feature entirely distinct histone acetylation patterns from both effector and memory T-cells.

For other genes, Escape samples initially shared features with one of the other groups during early infection, but later switched to a phenotype more similar to the other group. One example is the histone deacetylase *SIRT1*, for which Escape samples shared early low expression with Resolver samples and later expressed higher levels as in Chronic samples. This further substantiates the hypothesis that histone acetylation patterns in Escape T cells differ substantially from their antigen-cognizant counterparts in chronic infection. The deacetylase *SIRT1* is of special interest, as it is an important epigenetic modifier that acts as a repressor for the *TBX21* (T-bet) locus, a key transcription factor in T cell differentiation—making it an attractive target for immunotherapeutic intervention [25]. There are already small molecules under investigation that can reduce expression of another important T-cell transcription factor, *BATF*, while at the same time increasing *SIRT1*-mediated repression of *TBX21* [26], resulting in increased Tscm and Tcm populations [26]. In addition to an anti-inflammatory role through regulation of *TBX21*, *SIRT1* also plays an important role in regulation of mammalian metabolism, where it promotes fatty acid oxidation and gluconeogenesis [27].

Like *SIRT1*, a variety of other important metabolic regulators were expressed at consistently higher levels in Chronic samples throughout early and late acute infection, including *TBK1* and *BCOR*. *TBK1* facilitates induction of glycolytic enzymes as part of *HIF1A’s* positive regulation of glycolysis, which is particularly important in hypoxic conditions such as those encountered in the liver microenvironment. *HIF1A* itself is induced by TCR and IL-2 signaling and works to sustain Myc-dependent metabolic reprogramming [28,29] in response to TCR stimulation [28], which, in turn, is crucial to establish robust proliferation [30].

Interestingly, we also observed high expression levels of *BCOR* in Chronic and Escape samples. Unlike *TBK1*, the Bcl-6 co-repressor *BCOR* represses transporters and enzymes in glycolysis, dampening the metabolic program associated with effector differentiation [31,32,33,34]. This Bcl-6-dependent dampening is antagonized by T-bet, awarding this important T-cell transcription factor—which is required for specialization of Th1 cells by activating *IFNG* and *CXCR3* expression in a DNA-binding-dependent manner [35,36,37,38]—an important role in promoting glycolysis [32,33]. However, neither Bcl-6 nor T-bet were differentially expressed in our data set, suggesting that this specific pathway of regulating T-cell glycolysis might not be a key differentiating factor in HCV infection. However, we cannot rule out the possibility that another binding partner for *BCOR* regulates HCV-specific T-cells, as several other such binding partners have been described in the literature recently [39,40].

## 6. Metabolic Regulation in HCV-Specific CD8 T-Cells

While the standard differential gene expression analysis we employed in our recently published study identified several key differences between Acute and Chronic (and Escape) CD8 T-cell populations in genes linked to established processes in T-cell differentiation [20], supervised analytic approaches have clear limitations. This is especially true for complex cross-sectional human cohort studies that compare more than two groups across multiple conditions/timeframes, given heterogeneity in both genetic backgrounds and disease and sample variability. Such studies typically benefit from more complex semi-/unsupervised analytic approaches capable of identifying data patterns that cannot be known beforehand or that are masked by the chosen comparison groups.

Therefore, we further investigated CD8 transcriptional profiles using a semi-supervised weighted gene correlation network analysis approach (WGCNA) (see Figure 1). We combined early and late samples while preserving the overall sample group structure (Chronic, Escape, Resolver) to identify clusters (also called modules) of genes with highly correlated expression patterns across samples within each group. WGCNA is based on the assumption that genes with highly correlated expression patterns across samples that share a particular condition (e.g., Chronic HCV) represent genes with a common regulator (i.e., are co-regulated) in that condition. Identifying all such clusters of co-regulated genes for a particular condition forms a condition-specific module network, which can then be checked for module preservation across conditions and correlations of module-specific patterns with other sample traits (e.g., clinical parameters) [41].

By analyzing all three sample groups individually, we identified one specific set of genes related to metabolic pathways that were exclusively co-regulated in Chronic patients and associated mostly with two highly interconnected gene modules. Expression of genes within these modules was also strongly (negatively) correlated with time from infection, meaning that gene expression decreased with duration of viral persistence. Additional significant correlations were observed with patient age and sex, as well as a detectable presence of HCV-specific CD4 T-cells, with higher expression of genes in the metabolic signature observed in younger age, females, and presence of strong HCV-specific CD4 T-cell responses. As these patient traits are strongly associated with a higher likelihood of HCV resolution, this gene signature has potential to identify mechanistic links between sex, age, and T help and HCV control.

This Chronic-exclusive metabolic gene signature, which was marked by genes with high initial expression followed by rapid downregulation, was significantly enriched for genes related to oxidative phosphorylation. Downregulation of genes related to oxidative phosphorylation during early HCV infection seems paradoxical when considering that the electron transport chain presents a highly efficient means of energy production for a cell. However, activated and effector T-cells in general highly favor glycolysis as a means of energy production, while memory T-cells are more dependent on oxidative phosphorylation (OXPHOS) to meet their energetic demands [42,43]. Therefore, the observed downregulation in OXPHOS genes along with higher expression of glycolysis-promoting genes, such as *TBK1*, in the Chronic group suggests that perpetual activation might prevent these cells from activating a metabolic program that favors differentiation into memory cells.

Similar patterns of metabolic dysregulation in virus-specific CD8 T-cells also have been described in other human infections, such as HIV and, more recently, HBV as well as for lymphocytic choriomeningitis virus (LCMV) infection in mice [44,45,46]. While dysregulation signatures in both LCMV and HBV center around OXPHOS, the HBV signature is even closer to that in HCV, as the mouse signature also contained a strong signal of perturbed glycolysis not present in HBV or HCV. These results suggest that central features of CD8 T-cell metabolic dysregulation are shared across different models of T-cell exhaustion, with some variation in patterns and specific pathways of dysregulation that are most likely dependent on factors such as host species, infectious agent, and viral tropism.

## 7. Transcriptional Regulation of HCV-Specific CD8 T-Cells Beyond Metabolism

After identifying a distinct Chronic-exclusive signature of metabolic dysregulation, we examined underlying transcriptional regulators that might drive this change in metabolic programming. We hypothesized that transcriptional regulators would likely be annotated in the same modules as their targets if expression of the former linearly affected expression of the latter. Thus, we assembled information on known transcription factor-target interactions from multiple databases and intersected them with our weighted gene correlation network. In this way, we could estimate transcription factor-target coverage for each module (i.e., the number of interactions any given transcription factor had with genes annotated in the same module). Based on this information, we used the most highly connected transcription factors for each module of interest as a seed for a regulatory network, allowing connections between transcriptional regulators and targets not just within but also across closely connected modules to more realistically represent a functional regulatory network.

This approach resulted in a concise network of regulatory interactions that was largely Chronic-exclusive and provided links between transcriptional regulators and most genes in our metabolic dysregulation signature. However, the approach also extended interactions to genes more directly related to immune function and genes crucial to nucleosomal regulation of transcription that shared similar expression kinetics with drastically reduced expression as chronic infection progressed. The regulatory genes we identified to be the most likely drivers of these early transcriptional changes in chronic samples, which we used to seed the regulatory network, included various transcription factors, co-factors, and chromatin remodeling genes, including *CBX3*, *CREB1*, *FLI1*, *KDM5B*, *NELFE*, *RFX5*, *STAT1*, *STAT5A*, and *TP53*.

The seed for the regulatory network also included *IRF3* and *FLI1*, which were not annotated in the chronic gene modules of interest but were co-regulated with *CREB1*, *STAT1*, and *TP53* in a closely related Resolver module. These were introduced to allow the possibility that absence of certain regulators might be partly responsible for observed dysregulation in Chronic samples. While some of these genes are known transcriptional regulators of immune function, including *STAT1* and *STAT5A*, which are associated with effective IFN responses and T-cell memory differentiation [47], involvement in metabolic regulation is a less expected observation. For other genes, such as *TP53*, the opposite is true. *TP53* has a well-documented role in regulation of metabolism [48], but its immune-regulatory role has only recently been appreciated [49].

When examining the inferred transcriptional targets for these transcription factors in chronic HCV, we encountered not only most metabolic genes in the identified metabolic dysregulation signature, but also a wide range of genes related to nucleosomal regulation of transcription and chromatin modification. This included 21 genes coding for various histone subunits as well as many highly immune-relevant genes, such as *AKT1*, *CCL4*, *CCL5*, *CCR5*, *CD14*, *CD27*, *CD38*, *CD244*, *CX3CR1*, *ICAM1*, *ITGAL*, *ITGB2*, *ITGB7*, and *MYD88*. This further underlines the intersection between metabolic and nucleosomal regulation that was already suggested by some findings in the supervised gene expression analysis.

The reconstructed network also contained genes crucial to T-cell homing, migration, and adhesion. Among them was *ICAM1*, an important facilitator of intercellular adhesion, as well as its binding partner duo *ITGAL* and *ITGB2*, which together make up LFA-1. This is interesting because LFA-1 is not only a crucial regulator of T cells leaving the bloodstream and entering infected tissues but also has recently been reported to be an important factor in fine-tuning T cell effector function [50]. Another interesting finding in this context is that the Chronic network also featured *ITGB7*, but not its counterpart *ITGA4*. Both genes, which together form another important T-cell homing complex, were present in the Resolver network, where *ITGB2* was missing. Together this suggests that T cells in Chronic and Resolver samples might be subject to vastly different cell migration cues that affect their ability to successfully fight infection. Finally, the Chronic network also contained *CX3CR1*, another important lymphocyte homing factor whose presence and absence are indicative of effector and central memory T-cells, respectively [51]—again suggesting that rapid downregulation of *CX3CR1* might indicate these cells receive improper homing signals and fail to differentiate into a more long-lived anti-inflammatory phenotype.

Together, these data suggest that CD8 T-cells in Chronic HCV undergo rapid dysregulation of genes that are crucial for orchestration and direction of adaptive T-cell responses by impacting T-cell function, differentiation, and migration. Further, this dysregulation can be linked to known clinical predictors of HCV disease outcome and seems to be rooted in either simultaneous or rapidly sequential disruption of three integrally linked functional domains: immune function, metabolism, and nucleosomal regulation of transcriptional processes. While the exact order of regulatory events in early chronic HCV remains to be determined, it is likely to hold valuable information on the causal basis for T-cell exhaustion in HCV infection and in general.

Our study not only identifies pathways that are likely implicated in failure of the CD8 T-cell response to HCV infection but also highlights their high degree of interconnectedness—underlining the importance of tackling complex immunological problems with more holistic and integrative experimental and computational approaches. For example, large parts of cellular identity are encoded in the epigenetic landscape that produces a unique pattern of chromatin accessibility and modification, allowing establishment of different cell types from a shared genetic code. Epigenetic marks are important for both T cell reprogramming in response to antigenic stimulus [52,53,54] as well as their maintenance after stimulus withdrawal [55]. More recent work has further highlighted the existence of transcriptional-epigenetic profiles in the context of T-cell exhaustion in cancer and chronic viral infections as well as their role in establishment and reversibility of the exhausted phenotype [56,57,58,59,60]. In this context, it is important to note that T-cell exhaustion is a process that can be driven both by terminal differentiation of cells as well as by strong and prolonged antigen stimulation [61,62,63]. Further, exhausted cells exhibit profound heterogeneity with regard to their proliferative capacity [64,65,66,67,68,69], which might influence their capacity for exhaustion reversal.

In addition, it has become evident that metabolites have the capacity to regulate both epigenetic and transcriptional landscapes [70,71,72]. In many cases, metabolites are the substrates used to generate epigenetic modifications. Addition and removal of these modifications are, for the most part, catalyzed by enzymes whose activities are meditated by availability of substrates, cofactors, and allosteric regulators derived from metabolic pathways [73,74,75,76,77,78,79,80,81,82,83,84,85]. The fact that the epigenome is crucial to establish distinct cellular states [86,87,88] and critically involved in immune cell fate [89] makes the intersection and interaction of metabolism and epigenetics an exciting emerging field of study, particularly in the context of immunity.

## 8. Conclusions

We reviewed the first study utilizing HCV infection as a unique model disease for in-depth analysis of transcriptional regulation in human virus-specific T cells associated with distinct infection outcomes. We are convinced that this study will be followed by many others that will both deepen the findings from CD8 T-cells in early HCV infection as well as extend them to different infection scenarios that can be uniquely studied in HCV infection.

We are currently aware of multiple studies by different investigators using new generation technologies to more mechanistically examine metabolic dysregulation of HCV-specific CD8 T-cells and also analyze T cells before and after therapy. Additional studies will also better characterize HCV-specific CD4 T-cells and help understand different transcriptional states of HCV-specific CD8 T-cells at the site of infection, the liver. We are looking forward to publication of these results, with the expectation that the data will further establish the unique potential of studying human immunology in HCV infection.

Ongoing and future studies will also greatly benefit from constant expansion and refinement of the analytical tools used to understand such complex human data sets, including horizontal integration of distinct but interrelated processes such as metabolism and immune effector function. We will also learn about vertical regulation of T cell processes through epigenetic, transcriptional, translational, and post-translational processes. These insights will help understand cross-talk between T cells and different immune cell types or other components of the host immune response by integrating data from many different emerging studies [90] as well as using unbiased parallel analysis of different immune cells with technologies such as 10x or Seq-Well. There has never been a more exciting time to study HCV immunology.
